# Evaluation of a 5-HT_2B_ receptor agonist in a murine model of amyotrophic lateral sclerosis

**DOI:** 10.1038/s41598-021-02900-0

**Published:** 2021-12-08

**Authors:** Alizée Arnoux, Estelle Ayme-Dietrich, Stéphane Dieterle, Marc-Antoine Goy, Stephan Schann, Mélanie Frauli, Laurent Monassier, Luc Dupuis

**Affiliations:** 1 Mécanismes Centraux et Périphériques de la Neurodégénérescence, U1118, Inserm, UMR-S1118, CRBS, Université de Strasbourg, 1 rue Eugène Boeckel, 67000 Strasbourg Cedex, France; 2 Laboratoire de Pharmacologie et Toxicologie Neurocardiovasculaire, UR7296, Université de Strasbourg, 67000 Strasbourg, France; 3 Domain Therapeutics, 67400 Illkirch-Graffenstaden, France

**Keywords:** Diseases of the nervous system, Neurological disorders

## Abstract

Degeneration of brainstem serotonin neurons has been demonstrated in ALS patients and mouse models and was found responsible for the development of spasticity. Consistent with involvement of central serotonin pathways, 5-HT_2B_ receptor (5-HT_2B_R) was upregulated in microglia of ALS mice. Its deletion worsened disease outcome in the *Sod1*^G86R^ mouse model and led to microglial degeneration. In ALS patients, a polymorphism in *HTR2B* gene leading to higher receptor expression in CNS, was associated with increased survival in patients as well as prevention of microglial degeneration. Thus, the aim of our study was to determine the effect of a 5-HT_2B_R agonist : BW723C86 (BW), in the *Sod1*^G86R^ mouse model. Despite good pharmacokinetic and pharmacological profiles, BW did not ameliorate disease outcome or motor neuron degeneration in a fast progressing mouse model of ALS despite evidence of modulation of microglial gene expression.

## Introduction

Amyotrophic lateral sclerosis (ALS) is a neurodegenerative disease characterized by the joint involvement of lower motor neurons (LMNs), in the brainstem and spinal cord, and upper motor neurons (UMNs), in the motor cortex, leading to loss of body weight, progressive muscle paralysis and spasticity, and ultimately death within 2–5 years after onset. The pathophysiological mechanisms associated with ALS development are not well characterized, and to date no effective curative treatment is available. A subset of ALS cases are associated with a family history (familial ALS, fALS) and more than 30 different genes have been associated with fALS^1–3^. Among these, mutations in 5 genes are responsible of more than 60% of all cases: *C9ORF72*, *SOD1*, *TARDBP*, *FUS* and *TBK1*^4,5^. ALS pathophysiology involves multiple cell types beyond LMNs and UMNs. Specifically, ALS is associated with prominent activation of microglia, that contributes to motor neuron death. In the natural course of the disease, complex subpopulations of disease-associated microglia emerge whose respective roles in the disease process remain elusive^6^.

Spasticity is a cardinal symptom of ALS, and recent studies have demonstrated that degeneration of brainstem serotonin neurons, occuring in both mouse models and patients^7^, contributes to the development of this symptom^8^. We previously observed that the 5-HT_2B_ receptor (5-HT_2B_R) was upregulated at disease onset in the spinal cord of ALS mice^9^ and that deletion of *Htr2b,* encoding the 5-HT_2B_R, worsened disease outcome in the *Sod1*^G86R^ mouse model. Mechanistically, 5-HT_2B_R upregulation was restricted to CD11b + microglial cells, and deletion of *Htr2b* worsened microglial survival and altered gene expression of a number of microglial related pathways^9^. In ALS patients, a polymorphism in *HTR2B* gene leading to higher receptor expression in the central nervous system, was associated with increased survival in patients as well as prevention of microglial degeneration^9^. Thus, targeting the 5-HT_2B_R might influence microglial survival and gene expression, and, ultimately, be beneficial for human disease progression.

Here, we evaluated the preclinical potential of BW723C86 (BW), a full reference 5-HT_2B_R agonist in ALS. To evaluate the effect of 5-HT_2B_R stimulation in ALS, BW was selected due to two reasons: (1) its described selectivity for 5-HT_2_ receptors over the other serotonergic receptor families^10^ and (2) its non-amphetaminic chemical structure, since amphetaminic molecules such as fenfluramines, are anorexigens and loss of body weight is correlated to poor survival in ALS^11^. To this aim, we characterized the pharmacological profile of this compound, optimized its mode of delivery for diseases of the central nervous system, and characterized its efficacy and target engagement in *Sod1*^G86R^ mice, a model with fast progression of disease in which ablation of *Htr2b* accelerated disease^9^.

## Results

### BW is a 5-HT_2B_R ligand with weak binding to 5-HT_1A_ and 5-HT_1B_

We first sought to extensively perform BW pharmacological profiling using radioligand competition binding assay on a total of 106 targets (87 GPCR and 19 enzymes and transporters) at a single concentration of 10 µM of BW (Supplementary Table 2). At that dose, BW displayed a percentage of inhibition over 50% (Table 1; inhibition values indicated in grey-background cells) on 11 targets that were mainly serotonergic receptors (7 out of 11 targets). These 11 targets were further investigated in binding experiments (Supplementary Fig. 1). BW was most potent on 5-HT_2B_R with a pIC50 of 7.50 and a 100% inhibition of binding at the maximal concentration of 10 µM (Table 1). Two other serotonin receptors displayed around a log10 of potency difference for BW. 5-HT_1B_R, with 100% inhibition at 10 µM and a pIC50 of 6.74, and 5-HT_1A_R, with 95% percent inhibition at 10 µM and a pCI50 of 6.23. Binding on the verapamil site of calcium channel showed a 2.3 log10 difference, and pIC50 for the norepinephrine transporter (NET) could not be calculated (Table 1). On the 7 other targets, BW had a pIC50 difference between 1 and 2 log10 as compared to 5-HT_2B_R.Thus, BW is strongly binding to 5-HT_2B_R, yet might display off target activities on two other serotonin receptors, notably 5-HT_1A_R and 5-HT_1B_R.

**Table 1:** Pharmacological characterization of BW.

Target	Radiolabeled ligand	%Binding inhibition at 10 µM	Reference competitor (ref)	pIC50		%Binding inhibition at 10 µM		R^2^		Significant lack of fit?	
				Ref	BW	Ref	BW	Ref	BW	Ref	BW
5-HT2B	[3H]mesulergine	99.0	5-HT	7.68	7.50	94.50	103.54	0.97	0.97	No	No
5-HT1B	[125I]CYP(+ isoproterenol)	92.0	–	–	–	–	–	–	–	–	–
	[3H]5-CT	–	5-HT	8.56	6.74	103.54	101.39	0.99	0.92	No	Yes
5-HT1A	[3H]8-OH-DPAT	91.8	5-HT	8.42	6.23	104.54	95.26	0.97	0.95	No	No
DAT	[3H]BTCP	81.9	–	–	–	–	–	–	–	–	–
	[3H]WIN 35,428	–	GBR 12909	8.78	6.13	104.32	73.21	0.93	0.65	No	Yes
Ca^2+^ channel, ds	[3H]diltiazem	66.3	Diltiazem	4.79	6.11	116.63	35.80	0.92	0.66	Yes	No
5-HT7	[3H]LSD	89.5	5-CT	9.22	5.99	98.17	91.76	0.98	0.93	No	No
5-HT2C	[3H]mesulergine	97.8	5-HT	6.52	5.88	100.79	91.10	0.95	0.90	No	No
5-HT5A	[3H]LSD	54.6	Methiothepin	7.85	5.86	101.21	84.33	0.98	0.95	Yes	No
5-HT2A	[3H]ketanserin	92.0	Ketanserin	7.65	5.81	102.71	93.67	0.88	0.87	No	No
Ca^2+^ channel, vs	[3H]D888	75.5	Methoxyverapamil	6.56	5.21	112.06	51.29	0.87	0.74	No	No
NET	3H]nisoxetine	68.7	Desipramine	7.87	NC	100.21	74.42	0.97	0.78	No	Yes

Results of binding studies on the 11 most potent targets of BW.

BW is a full and fairly selective agonist on 5-HT_2B_ receptor.

### Optimization of BW dosage and route of administration

We then aimed at determining whether BW might have a pharmacological profile favourable for diseases of the central nervous system such as ALS and optimized its route and dose of administration for such indications.

**Figure 1:**
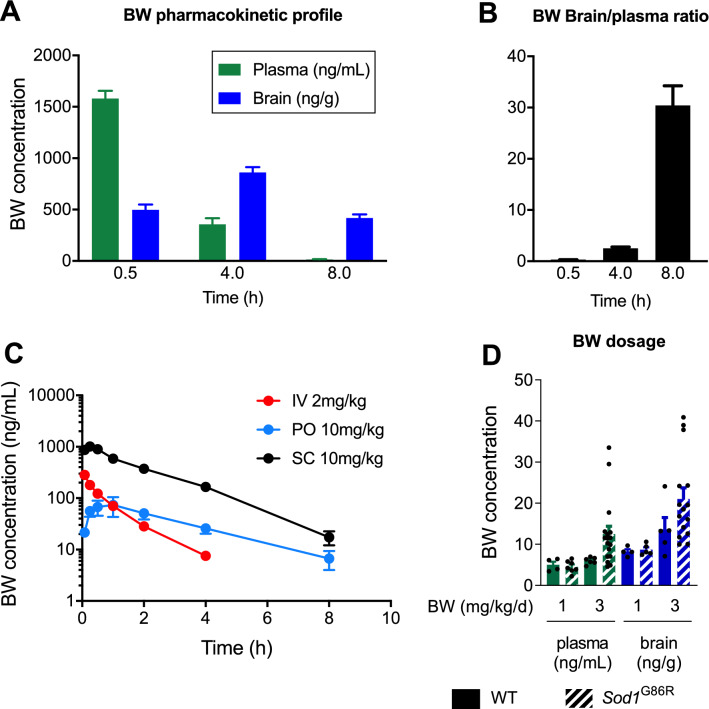
BW dosage and pharmacokinetic profile. (**A**) Kinetic concentration of BW in plasma and brain after a single IP injection of BW at 10 mg/kg. (**B**) Brain to plasma ratio of data presented on graph (**A**) (**C**) PK profile of BW after administration per os (PO), intravenous (IV) or subcutaneous (SC) in CD-1 mice. (**D**) Plasma and brain concentration of BW from our study animals treated with either 1 or 3 mg/kg/d.

We first determined whether BW might penetrate the CNS. To this aim, we compared plasma and brain concentrations after a single 10 mg/kg dose of BW injected intraperitoneally. Plasma concentration of 1580 ng/mL reaches its maximum at around 0.5 h, with a half-life of 1.10 h, and continuously decreases afterward to reach 14.4 ng/mL at 8 h (Fig. 1A). Interestingly, brain concentration of BW was higher at 4 h than at 0.5 h, reaching 860 ng/g and remained relatively high at 8 h post injection (417 ng/g). Thus, peripherally administered BW accumulated in the brain, as shown by the progressively increasing brain to plasma ratio (Fig. 1B).

We then compared BW plasma concentration over time, after intravenous (IV), *per os* (PO) and subcutaneous (SC) administrations. Subcutaneous administration was the most efficient route of administration tested, in regards to a C0 after IV injection of 354 ng/mL, with a Cmax of 1015 ng/mL and a bioavailability of 35% compared to 4.72% per os with a Cmax of 77 ng/mL (Fig. 1C and Table 2). At 24 h no compound could be detected after a single administration of BW (data not shown). Half-life values ranged from 0.93 h after IV injection to 2.55 h when administered PO, with 1.37 h for SC administration. In a similar manner, maximal plasma concentration was reached at 0.58 h and 0.19 h for respectively PO and SC administrations (Table 2).

**Table 2:** Pharmacokinetic properties of BW following different route of administration: per os (PO), intravenous (IV) or subcutaneous (SC).

	IV 2 mg/kg				PO 10 mg/kg				SC 10 mg/kg			
	n1	n2	n3	mean	n1	n2	n3	mean	n1	n2	n3	mean
C_0_/C_max_ (ng/mL)	406	290	366	354	36.5	61.5	132	77	935	1100	1010	1015
T_max_ (h)	–	–	–	–	0.25	0.50	1.00	0.58	0.08	0.25	0.25	0.19
T_1/2_ (h)	0.98	0.84	0.98	0.93	4.50	2.01	1.13	2.55	1.65	1.14	1.2	1.37
AUC_0-inf_ (ng.h/mL)	289	221	199	236	239	294	302	278	1938	2031	2246	2072
AUC_Extra (%)_	4.8	3.5	4.5	4.3	29.9	7.2	1.0	12.7	3.3	0.7	1.4	1.8
Bioavailability (%)				100%				4.7%				35%

Since BW appeared stable at 37 °C in vehicle for at least 2 months as determined by LC–MS (data not shown), we chose to use subcutaneous delivery using osmotic mini-pumps. To characterize the distribution and brain penetration of BW using this mode of administration, we measured BW in plasma and brain in our WT and *Sod1*^G86R^ mice after 4 weeks of administration. In these animals, BW administration allowed an average concentration of 4.6 ng/mL and 10.8 ng/mL at 1 and 3 mg/kg/d in the plasma, and brain concentrations respectively reached 8.5 ng/g and 19.2 ng/g in the brain (Fig. 1D). Interestingly, genotype appeared to modify BW metabolism, at least at 3 mg/kg/d of BW. Indeed, we observed a mean BW concentration of 6.0 ng/mL in the plasma and 13.7 ng/g in the brain of wild type mice, and 12.5 ng/mL in the plasma and 21.0 ng/g in the brain of *Sod1*^G86R^ mice (Fig. 1D). Thus, BW can be efficiently administered subcutaneously, through osmotic mini-pumps, leading to its penetration in the brain, even more in *Sod1*^G86R^ mice.

### BW neither modifies survival nor motor function in *Sod1*^G86R^ mice

**Figure 2:**
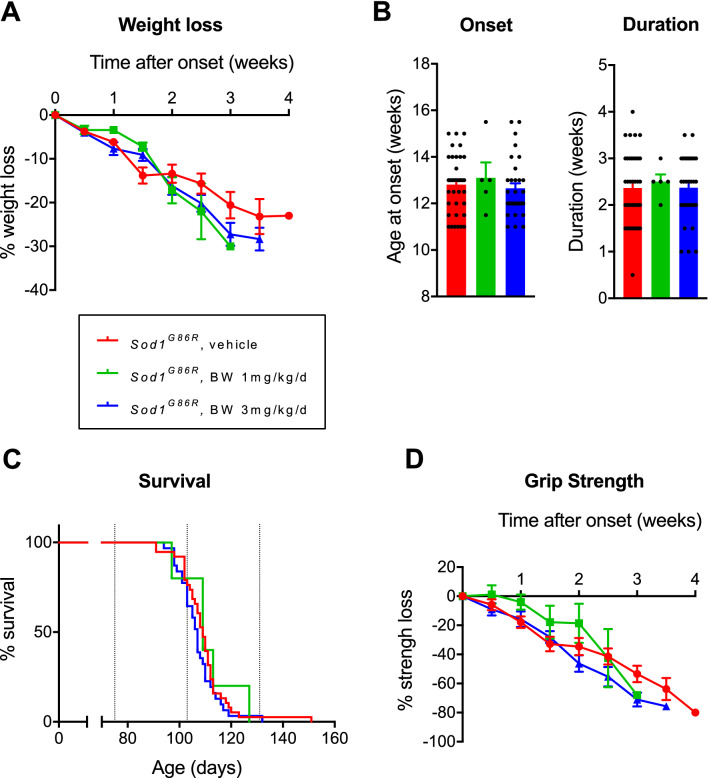
BW neither modifies survival nor motor function in *Sod1*^G86R^ mice. (**A**) Loss of body weight represented as percentage of loss after onset. Represented as mean +/− SEM. (**B**) Mice age at onset, defined as the peak of body weight and disease duration calculated as time between onset and death. Represented as mean +/− SEM. (**C**) Kaplan–Meier curve of survival. (**D**) Loss of muscular strength represented as percentage of loss after onset. Represented as mean +/− SEM. Groups are displayed as follow : *Sod1*^G86R^ in red (N = 38), *Sod1*^G86R^ treated with BW at 1 mg/kg/d in green (N = 5) and *Sod1*^G86R^ treated with BW at 3 mg/kg/d in blue (N = 31).

In order to evaluate the therapeutic potential of BW in ALS, we then treated transgenic mice expressing the G86R mutation of the murine *Sod1* gene (*Sod1*^G86R^ mice) with either vehicle (n = 38), 1 mg/kg/d (n = 5) or 3 mg/kg/d (n = 31) of BW. The drug was administered through osmotic mini-pumps, that were implanted subcutaneously at a presymptomatic age (75 days). Osmotic mini-pumps were collected at endstage, and mini-pump emptying was systematically checked. After mini-pump implantation, mice were longitudinally followed for body weight, grip strength and survival. Weight loss appeared unaffected by BW administration (Fig. 2A) with − 6.0, − 10.1 and − 8.6% weight loss per week for *Sod1*^G86R^ mice treated with vehicle (R^2^ = 0.96), 1 mg/kg/d (R^2^ = 0.93) or 3 mg/kg/d of BW (R^2^ = 0.98) respectively as determined using linear regression. Disease onset, defined as loss of 10% of maximal weight, (Fig. 2B) or disease duration (Fig. 2B) were unchanged upon 1 or 3 mg/kg/d of BW as compared to vehicle treated *Sod1*^G86R^ mice. The median survival was 109 days for *Sod1*^G86R^ mice treated with vehicle or 3 mg/kg/d of BW (*p* > 0.05 vs vehicle treated mice, log rank), and 107 days when treated with 1 mg/kg/d of BW (*p* > 0.05 vs vehicle treated mice, log rank), and results were similar in males (*p* > 0.05 vs vehicle treated mice, log rank) and females (*p* > 0.05 vs vehicle treated mice, log rank). Thus, BW did not modify survival of *Sod1*^G86R^ mice, whether in males or females (Fig. 2C).

**Figure 3:**
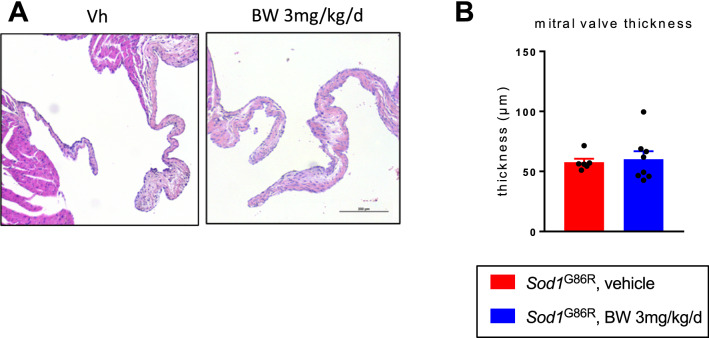
BW treatment does not alter valve thickness in *Sod1*^G86R^ mice. (**A**) Representative mitral valve morphology of *Sod1*^G86R^ mice treated with vehicle (left) or BW at 3 mg/kg/d (right) after hematoxylin/eosin staining. Objective × 10. (**B**) Average thickness (µm) of mitral valve of n = 6 and n = 8 *Sod1*^G86R^ mice treated with respectively vehicle (red) or 3 mg/kg/d of BW (blue).

All treated groups also displayed similar kinetics of muscle strength loss in similar proportions (Fig. 2D) (− 19.13, − 21.86 and − 23.26% respectively for *Sod1*^G86R^ mice treated with Vh (R^2^ = 0.98), 1 mg/kg/d (R^2^ = 0.86) or 3 mg/kg/d (R^2^ = 0.99) of BW respectively). Since BW did not affect survival or motor function of *Sod1*^G86R^ mice, we wanted to check that a potential beneficial effect of BW was not blunted by an adverse cardiovascular effect. To this aim, we measured mitral valve thickness using histology (Fig. 3). We did not observe any differences between *Sod1*^G86R^ mice treated with the vehicle (57.7 µm) and treated with 3 mg/kg/d of BW (60.2 µm). The lack of beneficial effect of BW on survival and motor function is not due to the developpement of valvulopathy nor cardiac failure. Thus, BW is not protective in *Sod1*^G86R^ mice independently of any potential cardiotoxicity.

### BW prevents upregulation of disease associated microglia gene expression in *Sod1*^G86R^ mice

**Figure 4:**
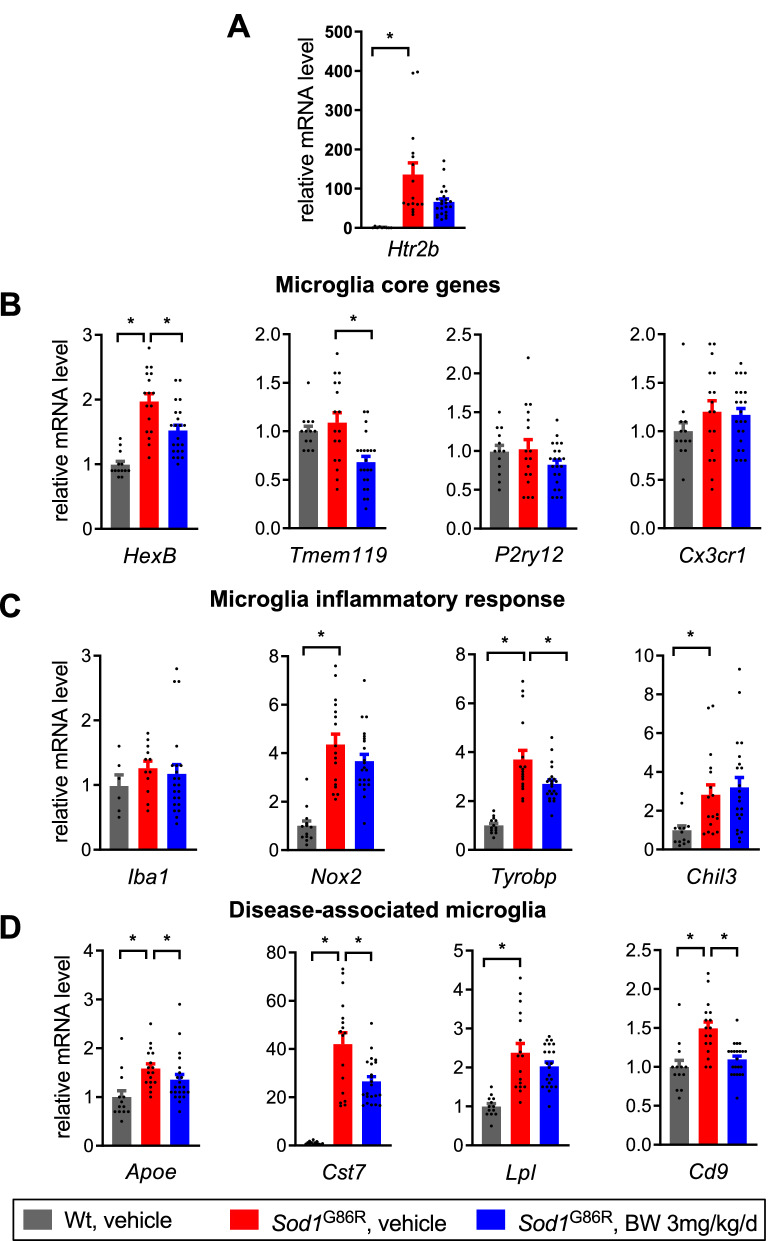
BW treatment modulates inflammation markers expression in spinal cord. (**A**) Expression of 5-HT_2B_ receptor, by RT-qPCR. (**B**) Expression of microglial marker of homeostasis and integrity. (**C**) Expression of microglial markers associated with inflammatory response. (**D**) Expression of microglial markers associated with DAM induction. All graphs presented as mean +/− SEM relative to the level of the wild-type control group. One-way ANOVA followed by Dunnett’s multiple comparison test. *p < 0.05.

**Figure 5:**
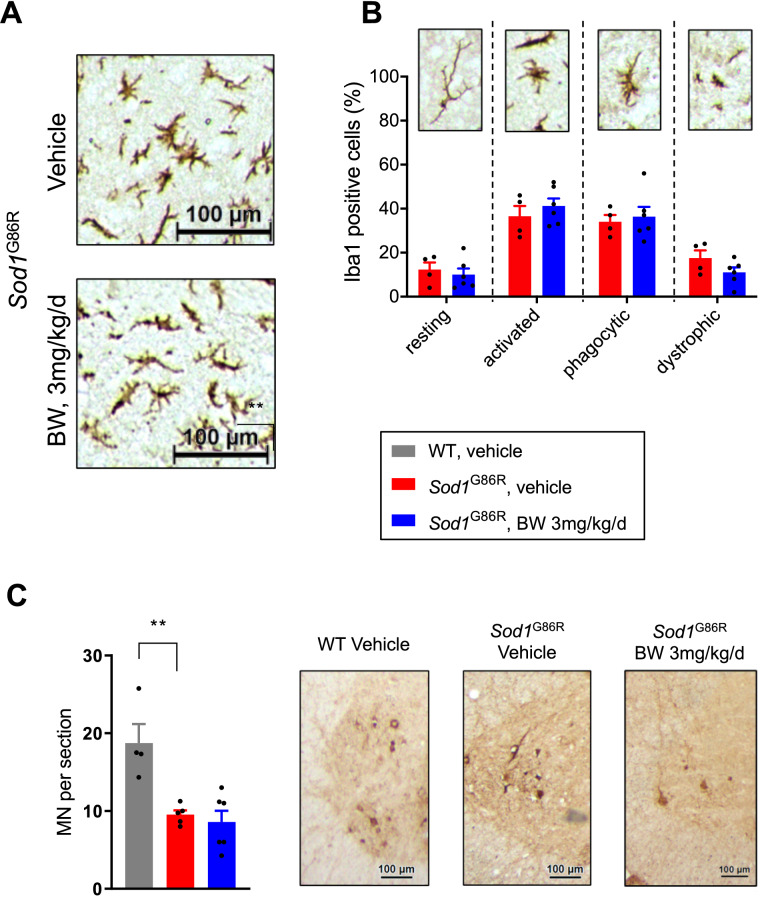
BW treatment does not modify microglia morphology and motor neuron degeneration in *Sod1*^G86R^ mice. (**A**) Representative Iba1 immunostaining of *Sod1*^G86R^ mice, treated with a vehicle (upper panel) or with 3 mg/kg/d of BW (lower panel). (**B**) Relative quantification of resting, activated, phagocytic and dystrophic Iba1 + cells. *Sod1*^G86R^ control animals are displayed in red (N = 4) and animals treated with 3 mg/kg/d of BW in blue (N = 6). Representative morphology of each microglial state is given on top of the graphic. (**C**) Motor neuron quantification in lumbar spinal cord. WT animals are displayed in grey (N = 4), *Sod1*^G86R^ control animals are displayed in red (N = 5) and animals treated with 3 mg/kg/d of BW in blue (N = 6). All graphs presented as mean +/− SEM. t-test. *p < 0.05. Representative ChAT immunostaining of wild type (left) and *Sod1*^G86R^ mice treated with either vehicle (middle) or BW at 3 mg/kg/d (right).

We then sought to characterize whether BW modulated microglial physiology and used RT-qPCR to measure microglia related genes in the cervical and thoracic spinal cord of animals treated with either vehicle or 3 mg/kg/d of BW. *Htr2B* was upregulated as expected in *Sod1*^*G86R*^ mice compared to littermates, with an approximate 100-fold increase at mRNA level (Fig. 4A). Expression of *Hexb*, a microglia core gene was upregulated in *Sod1*^G86R^ mice and this was partially prevented by BW (Fig. 4B). BW also partially prevented disease-associated upregulation of *Nox2* or *Tyrobp* (also known as DAP12) (Fig. 4C). Notably, BW blunted the upregulation of several disease-associated microglia markers, such as *Apoe, Cst7, Lpl* or *Cd9* (Fig. 4D)*.* We next wanted to see if these changes in gene expression were accompanied by modification of microglial activation in spinal cord of treated mice. Representative Iba1 immunostainings of a control mouse and a treated mouse are shown in Fig. 5A. Iba1 + cells were classified in 4 different activation states (resting, activated, phagocytic or dystrophic). No statistically significant difference in microglial morphology between groups was found regarding the proportion of each cell phenotypes (Fig. 5B). Importantly, BW treatment (3 mg/kg/d) did not modify the loss of motor neurons observed in *Sod1*^G86R^ mice as judged from counting ChAT positive cells in the ventral spinal cord (Fig. 5C). Thus, BW modifies microglia associated gene expression but does not appear to modify microglial morphological alterations or motor neuron degeneration in *Sod1*^G86R^ mice.

## Discussion

In this study, we provide a thorough characterization of BW723C86 pharmacology and pharmacokinetics, and characterize its effects in a mouse model of ALS. Our results indicate that BW is a selective 5-HT_2B_R ligand, ideal for targeting the 5-HT_2B_R in the CNS, and is able to modulate microglial gene expression in vivo. However, we did not observe increased survival or function upon treatment with BW in *Sod1*^G86R^ mice.

In the 1990s, there were few agonists that could discriminate between the different 5-HT_2_ receptor subtypes. The characterisation of BW723C86 followed a change in the classification of serotonin 5-HT_2_ receptors, bringing together the three subtypes 5-HT_2A_, 5-HT_2B_ and 5-HT_2C_ receptors within the same family by sequence homologies^12^. A series of ligands with affinity for 5-HT_2_ receptors, including BW723C86, were therefore re-characterised to compare their selectivity between the three receptor subtypes, in order to study their potential therapeutic interest in psychiatric indications^12^. BW723C86, one tryptamine analogue, showed some selectivity for the 5-HT_2B_ receptor, and acted as a potent agonist in rat stomach fundus, while displaying much lower potency at 5-HT_2A_ and 5-HT_2C_ receptors^13^. This selectivity is not specific to the rat and was also demonstrated in vitro on human receptors by Porter et al., thus providing a useful tool for the study of central 5-HT_2B_R^14^. During these years of debate around the comparison of agonist radioligand binding at all three human 5-HT_2_ receptors subtypes^10^, the assessment of binding to other serotonergic receptors has been underestimated.

A first important result of our study is a detailed characterization of BW pharmacology. We bring here two novel notions on this drug. First, BW, once presented as a full reference 5-HT_2B_R agonist displays non negligible binding to other serotonin receptors, specifically 5-HT_1B_ and 5-HT_1A_ receptors in a ratio of around one log. The binding of BW723C86 to the 5-HT_1B_ receptor bears significant interest as these two receptors are already used as targets in the acute and preventive treatment of migraine^15^. It is however important to note that our current study did not investigate the functional effect of BW723C86 on the 5-HT_1B_ receptor, and that this requires further study. A second important pharmacological observation we obtained was that BW723C86 surprisingly lacked binding to the serotonin transporter (SERT), while potently binding the norepinephrine (NET) and dopamine (DAT) transporters. Thus, BW723C86 is not a SERT substrate, unlike 5-HT_2B_ agonists displaying an amphetamine-like structure. This has important consequences on its mode of actions since lack of SERT binding suggests that BW723C86 should not promote 5-HT release, and that its effects are most-likely receptor-dependent and serotonin-independent. In addition to these pharmacological properties, its pharmacokinetic profile indicates that the subcutaneously administration should be preferred for chronic treatments, due to its poor oral bioavailability, and allows significant diffusion and accumulation in the central nervous system.The higher intracerebral concentration of BW in *Sod1*^G86R^ mice than littermates, at 3 mg/kg/d associated with the increase in plasma BW concentration in *Sod1*^G86R^ mice suggests a decrease in metabolism or its elimination, that could occur through either inhibition of enzyme or efflux transporter responsible for BW bioavailability. The pharmacokinetic parameters involved in the variability of the effect of BW723C86 should therefore be studied. In all, our study provides surprising insights into the pharmacology and pharmacokinetics of a classically used full reference 5-HT_2B_R agonist, that could be of importance for further indications involving CNS 5-HT_2B_R.

Previous results from our team identified the 5-HT_2B_R as a potential therapeutic target in ALS, and building on our new pharmacological knowledge, we evaluated the therapeutic potential of BW in a mouse model of ALS. We focused on the *Sod1*^*G86R*^ mouse model for several reasons: first, this mouse line is fast progressing, reaching endstage within 3 to 4 months of age, associated with explosive microgliosis at endstage, similar to the more commonly used *SOD1*^*G93A*^ model. Second, all previous research on the 5-HT_2B_R^9^ has been performed on this model, rendering it ideal for these studies. Indeed, several lines of evidence previously linked serotonin, and specifically the 5-HT_2B_R to ALS. First, serotonin levels appeared decreased in ALS patients in platelets, which correlated with survival^16^, while central serotonergic neurons degenerate both in patients and mouse models, and contribute to ALS-related spasticity^7^. In the *SOD1*^G37R^ conditional mice, we further showed that rescue of serotonergic neurons was able to rescue spasticity^8^, yet exacerbating the disease. A candidate receptor underlying serotonin effects in ALS is the 5-HT_2B_R. Indeed, *Htr2b* expression was upregulated in the spinal cord of *Sod1*^G86R^ mouse model of ALS at pre-symptomatic stage^9^, and its deletion worsened disease outcome in this model. Upregulation of the 5-HT_2B_R expression in *Sod1*^G86R^ was found exclusively on CD11b + mononuclear phagocytes, presumable microglia, and *Htr2b* deletion increased microglial degeneration^9^. Since deletion of *Htr2b* worsened ALS-like symptoms, stimulation of the 5-HT_2B_R appeared as an attractive therapeutic target in ALS.

BW however did not provide protection in *Sod1*^G86R^ mice, on disease progression, survival or motor function at 1 or 3 mg/kg/d. We used here both sexes and performed studies at two doses, including one with a large number of animals, which suggest that our negative result is not due to lack of power or insufficient dosage. 5-HT_2B_R agonists are well-known to induce cardiac side effects in the form of valvulopathy development^17^. We analyzed mitral valve thickness between *Sod1*^G86R^ treated with the vehicle or BW at 3 mg/kg/d to assess if the potentially beneficial effect of BW on ALS was blunted by any adverse cardiac effect. No valvulopathy was observed in BW treated mice as attested by mitral valve thickness that was similar in both groups. Thus the lack of effect of BW on ALS is not due to the parallel development of valvulopathy. Indeed, the combination of our pharmacokinetic studies and the observation of treatment related alterations in gene expression demonstrate that the drug reached the spinal cord and targeted microglial physiology as expected. Administration of BW blunted upregulations of several markers of disease associated microglia, that are expected to rise with disease progression in ALS mice^18,19^. It is noticeable however that the transcriptional response observed in microglial gene expression is, at least for a subset of genes, different in our control groups than in our previous study^9^, in particular for *Tmem119* or *Cx3cr1* that were not upregulated in *Sod1*^G86R^ mice in this study. A possible explanation is that mice between both studies do not share the same genetic background due to backcrossing of the *Htr2b* null allele in the 129 Sv/PAS background. Indeed, genetic background is a major source of microglial heterogeneity as shown recently^20^.

Strikingly, the transcriptional response observed in microglial gene expression is, at least for a subset of genes, similar to what observed in *Htr2b* knock-out mice, while we aim here at stimulating the 5-HT_2B_ receptor. For instance, *Hexb, Tmem119, Nox2* or *Tyrobp* were similarly downregulated in *Sod1*^G86R^ mice with *Htr2b* ablation or BW treatment. This might be caused by the chronic activation of the 5-HT_2B_R in BW treated mice, leading to its desensitization hence loss of its function, and could be a major limitation in our interpretation of this negative result.

Our study is however limited by the use of the *Sod1*^G86R^ model, a severe model of ALS with explosive microglial activation in end stage and we cannot exclude that BW might provide protection in a slowly progressive mouse model. Another limitation is that we restricted the current study to a SOD1-based model, while ALS cases caused by *SOD1* mutations account for less than 1% of total ALS cases. In addition, both 5-HT_1B_ and 5-HT_1A_ receptors are present in the CNS and are involved in mechanisms such as anxiety, depression, migraine. Notably, 5-HT_1B_ receptor is involved in the regulation of food intake via the melanocortin pathway. Its activation leads to an hypophagic effect, in addition to increase POMC and CART gene expression and decrease orexin gene expression^21^. Energetic balance is a crucial feature in ALS and is positively correlated to survival in both mice and patients, and the melanocortin pathway is impaired in both mouse models and ALS patients^22^. On the contrary, activation of 5-HT_1A_ presynaptic receptors might have a positive impact on behaviours related to degeneration of serotonergic neurons like depression, by triggering serotonin release. In our study we did not find differences in body weight loss between our control and treated groups, but we cannot exclude an adverse effect linked to the activation of the 5-HT_1B_ receptor, and subsequent hypophagic behaviour, as food intake was not measured. Further research is thus warranted using other 5-HT_2B_R agonists and/or other ALS models to reach a definitive answer regarding the potential of BW as a treatment to ALS.

Overall, while our study was not able to demonstrate a therapeutic potential for BW723C86 in ALS, it provides a better characterization of pharmacology and pharmacokinetics of this drug, and suggests that disease-associated microglia emergence partially depends on the 5-HT_2B_R. The latter conclusion might be of interest for the other neurodegenerative conditions associated with these abnormal microglial populations, including Alzheimer’s disease.

## Methods

### Mice

*Sod1*^G86R^ mice on FVB/N background were produced by rapid lineage extension at Charles River facility, France. The resulting animals were F1 hybrid FVB/NRj and FVB/NCrl. During experimentation, mice were housed at the animal facility of the Faculty of Medicine of the University of Strasbourg, in a conventional sanitary status. They had food and water ad libitum and a 12 h/12 h of light/dark cycle. Seventy-five-days-old *Sod1*^G86R^ mice and their wild-type (WT) littermates were randomized into groups treated with either 1 mg/kg/d or 3 mg/kg/d of BW, or vehicle (Vh). We included both females and males in equal proportions. Treatments were administered by subcutaneous perfusion with Alzet® osmotic pumps model 1004 (Alzet®, California, USA; provided by Charles River, France). Implantations were performed under 1.5% isoflurane anaesthesia (Iso-Vet, Piramal Healthcare, UK). Mice were sacrificed at end-stage by lethal intraperitoneal injection of pentobarbital (Doléthal 120 mg/kg, Vetoquinol, France). Spinal cord, brain and blood samples were then collected. All animal care and procedures were in accordance with institutional guidelines and European regulations. All procedures were authorized by the local ethic committee (CREMEAS, Comité Régional d'Ethique en Matière d'Expérimentation Animale de Strasbourg) and the relevant French ministry under the authorization #13598- 201802160948218, in accordance with the ARRIVE guidelines.

### Treatment

1-[5-(thiophen-2-ylmethoxy)-1H-indol-3-yl]propan-2-amine (BW723C86) hydrochloride was synthesized by Wuxi (Wuxi AppTec, China). The vehicle is 20% DMSO in distilled water. The pumps were replaced every month until sacrifice at end-stage to ensure continuous delivery of drugs. Pumps were activated 48 h prior implantation by putting them in saline water at 37 °C to ensure treatment continuity. All experiments were performed blinded for the observer.

### Muscular strength assessment and survival

Mice were visually monitored daily for any potential ethical issue. Body weight and muscular strength were monitored twice a week. Muscular strength was assessed using a gripmeter test (ALG01, Bioseb, France). Results are the mean of three successive measures and were normalized by body weight. Onset of the pathology was calculated as the time between birth and peak of body weight. Duration of the disease is the time between onset and death of the mouse. End-stage was defined as the inability of the animal to turn around after 3 s placed on the back, and subsequent euthanasia followed. Muscular strength and weight loss are presented as percent loss from disease onset. Linear regressions were calculated using the least squares method using GraphPad Prism version 7.05 for Windows (GraphPad Software, La Jolla California USA, www.graphpad.com RRID: SCR_002798). Survival is presented as Kaplan–Meier curves.

### Histology

Lumbar spinal cord was dissected and cryopreserved before OCT inclusion. Samples were cut with a cryostat (Leica CM30505) at 20 µm of thickness. Iba1 (1:100, Sigma cat#SAB2702364, RRID: AB_2820253) and ChaT (1:100, Millipore cat#AB144P) immunostainings were performed using DAB peroxidase technique to assess microglial integrity and motor neuron degeneration. Images were acquired using a Leica DM750 microscope at × 10 magnification. Cells were counted in the ventral horn in a standardized zone with n = 4–10 images per animal, and subsequently classified using FIJI software (RRID: SCR_002285)^23,24^.

After dissection and rinse with PBS 1X, hearts were fixed for 72 h in formaldehyde 4% solution. After paraffin inclusion, cuts of 4 µm thickness were made. Hematoxylin/eosin staining was performed on these cuts. Inclusion and staining were performed by the department of histopathology and embyology of the mouse clinical institute (ICS, Illkirch-Graffenstaden). Valve imaging was performed with a Leica DFC425C camera mounted on a microscope Leica DM750, at × 10 magnification (cat# 506228, 10x/0.25 HI PLAN ∞/−), with LAS V4.8 software (Leica Microsystems). Analyses were performed with FIJI (RRID: SCR_002285) and GraphPad Prism version 7.05 for Windows (GraphPad Software, La Jolla California USA, www.graphpad.com RRID: SCR_002798) softwares. Average thickness was determined by rapporting the area on the length of the leaflet.

### RT-qPCR

Total RNA was extracted from the cervical and thoracic spinal cord of end stage mice using TRIzol (Invitrogen). Reverse transcription was performed using 1 μg of RNA and the iScript cDNA synthesis kit (BioRad). IQ SYBR green Supermix (BioRad) was used to perform RT-PCR. Data were normalized with GeNorm software (RRID: SCR_006763)^25^. Three reporter genes were used for normalization: POL2, TBP and Actin. Oligonucleotide primers are provided in Supplementary Table S1.

### Dosage and pharmacokinetic studies

Pharmacokinetic analyses of BW were performed by Wuxi (Wuxi AppTec, China). In the first assay, we evaluated the penetration capacity of BW in the CNS, by comparing its plasma and brain distribution, after intraperitoneal infusion at 10mk/kg in CD-1 mice (n = 3). Plasma and brain concentrations were measured at 0.5, 4 and 8 h after injection. Brain to plasma ratio (B/P ratio) is calculated as brain /plasma concentrations for each time point. In the second assay, we compared 3 modes of administration: intravenous (IV), per os (PO) and subcutaneous (SC). Respective doses were as follow: 2 mg/kg IV and both PO and SC at 10 mg/kg, on n = 3 mice per group. Pharmacokinetic data are presented in Table 1. Plasma assays followed a single dose administration and were performed at different time points: 0.083, 0.25, 0.5, 1, 2, 4, 8 and 24 h. Brain and plasma concentrations of BW were determined by LC–MS/MS by Eurofins (Eurofins ADME Bioanalyses, France). These assays were performed on end-stage *Sod1*^G86R^ mice and their littermates included in the survival study. Brain dosages were performed on n = 5 WT and n = 15 *Sod1*^G86R^ mice treated with 3 mg/kg/d of BW, and on n = 4 WT and n = 5 *Sod1*^G86R^ mice treated respectively with 1 mg/kg/d of BW. Plasma dosages were performed on n = 6 WT and n = 17 *Sod1*^G86R^ mice treated with 3 mg/kg/d of BW, and on n = 4 WT and n = 7 *Sod1*^G86R^ mice treated respectively with 1 mg/kg/d of BW.

### Pharmacological studies

A broad pharmacological study of BW on 106 targets, listed in Supplementary Table S2, was performed by Eurofins (Eurofins Cerep, France). This study included binding assays (87 GPCR targets), measured as % of binding inhibition of a reference radioligand, as well as uptake and enzyme assays (19 targets), the latter measured as % inhibition of control enzyme activity. A single dose of 10 µM of BW was tested in duplicate, and any target on which the compound presented a binding inhibition higher than 50% was selected for the following dose–response study. This second binding study was thus performed on a restricted list of 11 targets that were top ranked in the previous binding study. It was performed by radioligand binding competition by Euroscreen (Euroscreen FAST, Belgium). The following nanomolar concentrations were tested in duplicate: 0.001, 0.01, 0.1, 1, 10, 100, 1000, 10,000. A detailed list of radiolabelled ligands and reference compounds are given in Table 2. Dose–response curves were fitted with the least squares method. These results and R^2^ values are given in Table 2. IC50 results are shown as pIC50, corresponding to the negative log10 of IC50 in molar. All these analyses were performed using GraphPad Prism version 7.05 for Windows (GraphPad Software, La Jolla California USA, www.graphpad.com RRID: SCR_002798).

### Statistical analysis

All data are represented as mean +/− standard error of the mean (SEM). The number of animals for each experiment may vary and is indicated in the legends of the figures. Significance threshold was *p* < 0.05. Survival curves comparison was performed with Log-rank (Mantel–Cox) test. When performed, statistical test’s choice was based on the result of the D’Agostino–Pearson normality test. If the considered values followed a Gaussian distribution, a parametric test was performed (t-test for a 2-group comparison, one-way ANOVA followed by Dunnett’s multiple comparison for multiple group comparison), else a non-parametric test was performed (Mann–Whitney for a 2-group comparison, Kruskal–Wallis followed by Dunnett’s multiple comparison for multiple group comparison). All statistical analyses were performed using GraphPad Prism version 7.05 for Windows (GraphPad Software, La Jolla California USA, www.graphpad.com RRID: SCR_002798).

### Data exclusion

A non-negligible number of *Sod1*^G86R^ animals were excluded a posteriori due to the following reasons: (1) Animals declaring onset before the beginning of the treatment (respectively for Vh, BW1 and BW3 groups: 8, 2 and 4 mice), and (2) animals declaring onset after 112 days as it was not a treatment-induced delay and the numbers were too small to treat them as subgroups (respectively 7, 2 and 6 mice). Their exclusion did not modify results.

## Supplementary Information

Supplementary Information.

## References

[1] Brown RH, Al-Chalabi A (2017). Amyotrophic lateral sclerosis. N. Engl. J. Med..

[2] van Es MA (2017). Amyotrophic lateral sclerosis. Lancet.

[3] Taylor JP, Brown RH, Cleveland DW (2016). Decoding ALS: From genes to mechanism. Nature.

[4] Chiò A (2014). Genetic counselling in ALS: Facts, uncertainties and clinical suggestions. J. Neurol. Neurosurg. Psychiatry.

[5] Freischmidt A (2015). Haploinsufficiency of TBK1 causes familial ALS and fronto-temporal dementia. Nat. Neurosci..

[6] Clarke BE, Patani R (2020). The microglial component of amyotrophic lateral sclerosis. Brain.

[7] Dentel C (2013). Degeneration of serotonergic neurons in amyotrophic lateral sclerosis: A link to spasticity. Brain.

[8] El Oussini H (2017). Degeneration of serotonin neurons triggers spasticity in amyotrophic lateral sclerosis. Ann. Neurol..

[9] El Oussini H (2016). Serotonin 2B receptor slows disease progression and prevents degeneration of spinal cord mononuclear phagocytes in amyotrophic lateral sclerosis. Acta Neuropathol..

[10] Knight AR (2004). Pharmacological characterisation of the agonist radioligand binding site of 5-HT(2A), 5-HT(2B) and 5-HT(2C) receptors. Naunyn Schmiedebergs Arch. Pharmacol..

[11] Dupuis L, Pradat P-F, Ludolph AC, Loeffler J-P (2011). Energy metabolism in amyotrophic lateral sclerosis. Lancet Neurol..

[12] Baxter G, Kennett G, Blaney F, Blackburn T (1995). 5-HT2 receptor subtypes: A family re-united?. Trends Pharmacol. Sci..

[13] Baxter GS (1996). Novel discriminatory ligands for 5-HT2B receptors. Behav. Brain Res..

[14] Porter RH (1999). Functional characterization of agonists at recombinant human 5-HT2A, 5-HT2B and 5-HT2C receptors in CHO-K1 cells. Br. J. Pharmacol..

[15] Barbanti P, Aurilia C, Egeo G, Fofi L, Palmirotta R (2017). Serotonin receptor targeted therapy for migraine treatment: An overview of drugs in phase I and II clinical development. Expert Opin. Investig. Drugs.

[16] Dupuis L (2010). Platelet serotonin level predicts survival in amyotrophic lateral sclerosis. PLoS ONE.

[17] Ayme-Dietrich E (2017). The role of 5-HT2B receptors in mitral valvulopathy: Bone marrow mobilization of endothelial progenitors. Br. J. Pharmacol..

[18] Deczkowska A (2018). Disease-associated microglia: A universal immune sensor of neurodegeneration. Cell.

[19] Keren-Shaul H (2017). A unique microglia type associated with restricting development of Alzheimer’s disease. Cell.

[20] Yang HS (2021). Natural genetic variation determines microglia heterogeneity in wild-derived mouse models of Alzheimer’s disease. Cell Rep..

[21] Nonogaki K (2007). Fluvoxamine, a selective serotonin reuptake inhibitor, and 5-HT2C receptor inactivation induce appetite-suppressing effects in mice via 5-HT1B receptors. Int. J. Neuropsychopharmacol..

[22] Vercruysse P (2016). Alterations in the hypothalamic melanocortin pathway in amyotrophic lateral sclerosis. Brain.

[23] Schindelin J (2012). Fiji: An open-source platform for biological-image analysis. Nat. Methods.

[24] Schneider CA, Rasband WS, Eliceiri KW (2012). NIH Image to ImageJ: 25 years of image analysis. Nat. Methods.

[25] Vandesompele J (2002). Accurate normalization of real-time quantitative RT-PCR data by geometric averaging of multiple internal control genes. Genome Biol..

