# Supplementary material for: Evaluation of a 5-HT2B receptor agonist in a murine model of amyotrophic lateral sclerosis
Source: Sci Rep. 2021 Dec 8;11:23582. doi: 10.1038/s41598-021-02900-0 (PMC8654833; doi:10.1038/s41598-021-02900-0)
Supplement: Supplementary file 1 — Supplementary Information. [file 41598_2021_2900_MOESM1_ESM.pdf]

Arnoux et al., Supplementary Figure S1

Binding experiments on the 11 most potent targets of BW.

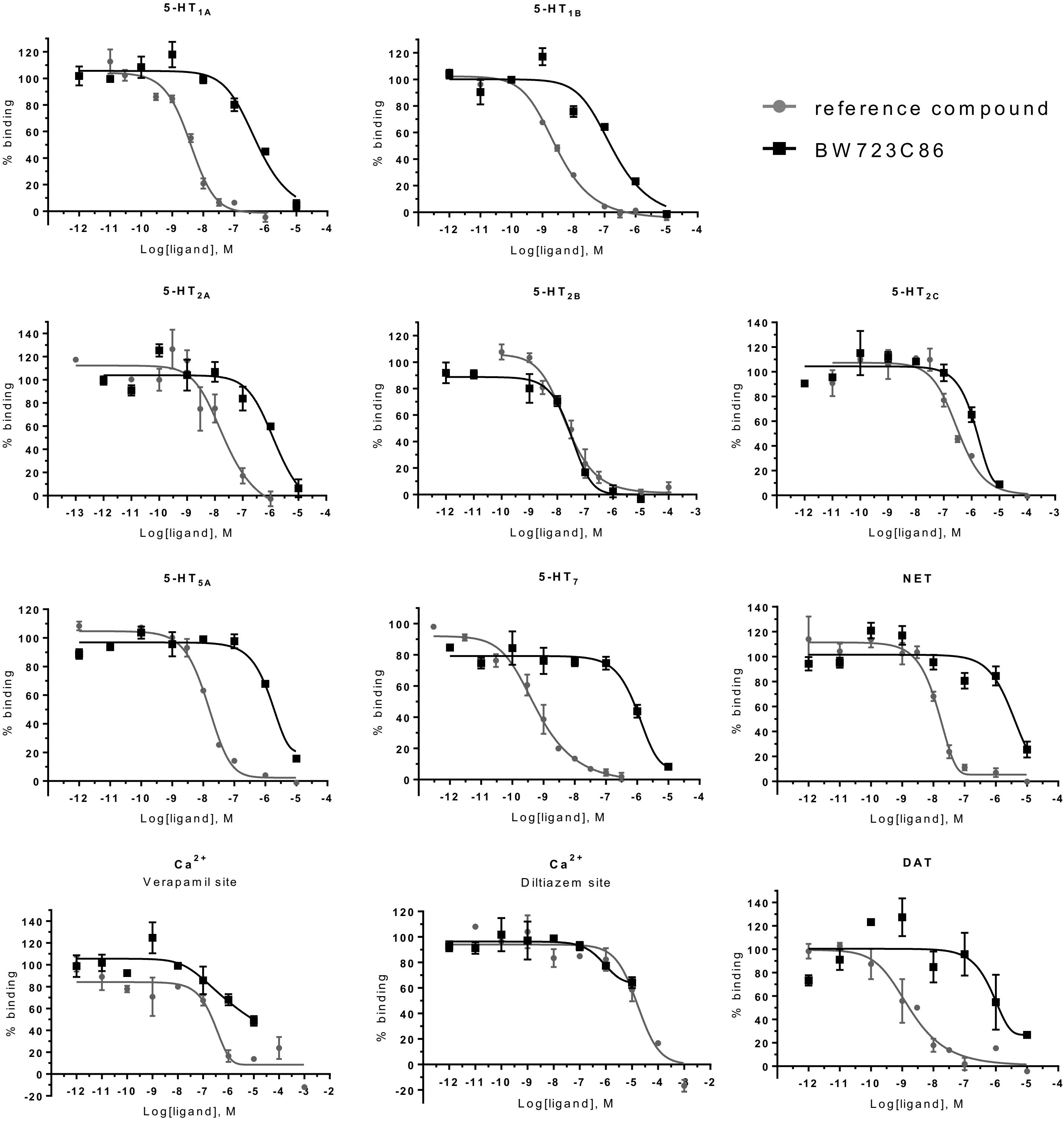

Supplementary Table S1 : Oligonucleotide primers used in our RT-qPCR experiments

target	5'- forward -3'	5'- reverse -3'
POL2	GCTGGGAGACATAGCACCA	TTACTCCCCTGCATGGTCTC
TBP	CCAATGACTCCTATGACCCCTA	CAGCCAAGATTCACGGTAGAT
Actin	CCACCAGTTCGCCATGGAT	GGCTTTGCACATGCCGGAG
Nox2	ACACTGACCTCTGCTCCTGA	AGGCATCTTGGAACTCCTGC
YM1	GAAGGACCATGGAGCAGCTT	GGGGCACCAATTCCAGTCTT
HexB	GGACTTCAGCATCGACCACA	GTAATATCGCCGAAACGCCTC
Iba1	AGCTTTTGGACTGCTGAAGG	CAGCTCTAGGTGGGTCTTGG
ApoE	CTTCTGGGATTACCTGCGCT	CTCCATCAGTGCCGTCAGTT
Dap12	TGACTCTGCTGATTGCCCTG	GGCGACTCAGTCTCAGCAAT
P2ry12	ACCACCCCTGTTTTTCCAGTT	AGCCTTGAGTGTTTCTGTAGGG
Cx3cr1	AGCTCACGACTGCCTTCTTC	GGTTGTTCATGGAGTTGGCG
Tmem119	ACCCAGAGCTGGTTCCATAG	GAGTGACACAGAGTAGGCCA
Lpl	TGCCCGAGGTTTCCACAAAT	AGCAGTTCTCCGATGTCCAC
Cst7	AGTCCCATGTCAGCAAAGCC	GTTCGGCCGATTTTCACCTC
CD9	TGGGGCTATACCCACAAGGA	GCTTTGAGTGTTTCCCGCTG

POL2 : Polymerase 2 ; **TBP** : TATA box-binding protein ; **Nox2** : NADPH oxidase 2; **YM1** : Chitinase 3-like 3 ; **HexB** : Hexosaminidase B ; **ApoE** : Apolipoprotein E ; **P2ry12** : Purinergic Receptor P2Y12 ; **Cx3cr1** : CX3C chemokine receptor 1 ; **Tmem119** : Transmembrane Protein 119 ; **Lpl** : lipoprotein lipase ; **Cst7** : Cystatin-F

Supplementary Table S2 : Exhaustive list of the 106 targets in binding assays

target list	
5-HT transporter	inducible NOS
5-HT1A	IP (PGI2)
5-HT1B	KATP channel
5-HT2A	M1
5-HT2B	M2
5-HT2C	M3
5-HT3	M4
5-HT5a	MAO-A
5-HT7	MAO-B recombinant enzyme
A1	MMP-1
A2A	MMP-2
A2B	MMP-3
A3	MMP-7
adenosine transporter	N neuronal α4β2
AMPA	Na+ channel (site 2)
B1	NK1
B2	NK2
BB1	NK3
BZD	NMDA
Ca2+ channel (L, dihydropyridine site)	NMU2
Ca2+ channel (L, diltiazem site)	NOP (ORL1)
Ca2+ channel (L, verapamil site)	norepinephrine transporter
Ca2+ channel (N)	NTS1 (NT1)
CB1	opioid (non-selective)
CB2	P2X
CCK2 (CCKB)	P2Y
CGRP	PAC1 (PACAP)
Cl- channel (GABA-gated)	PCP
constitutive NOS (endothelial)	PDE4A1A
COX1	PDE4B1
COX2	PDE4D2
CRF1	PR
D2L	sigma (non-selective)
D2S	sigma 1
dopamine transporter	sigma 2
elastase	SKCa channel
EP1	sPLA2
EP4	sst4
ERK2 (P42mapkk)	TNF-α
ET1	TRH1
ETB	tyrosine hydroxylase
GABA transaminase	VPAC1 (VIP1)
GABA transporter	xanthine oxidase / superoxide O2- scavenging
GABAA1	Y1
GABAB(1b)	Y2
GAL1	α1
GAL2	α1D
glycine (strychnine-insensitive)	α2A
glycine (strychnine-sensitive)	α2C
H1	β1
H2	δ (DOP)
H3	κ (KOP)
I2	μ (MOP)
